# Assessment of impact of land use change on the wetland in Makhitha village, Limpopo province, South Africa

**DOI:** 10.4102/jamba.v11i2.693

**Published:** 2019-07-03

**Authors:** Mpho D. Phethi, Jabulani R. Gumbo

**Affiliations:** 1 Department of Ecology and Resource Management, University of Venda, Thohoyandou, South Africa; 2 Department of Hydrology and Water Resource Management, University of Venda, Thohoyandou, South Africa

**Keywords:** Wetland Degradation, Subsistence Farming, Environmental Awareness, Poverty, Land Use

## Abstract

Wetlands are essential for the natural function of an ecosystem, by regulating floods and remaining as a source of water supply. However, land use changes are the main forces behind wetland deterioration worldwide, including South Africa. In this article, we report on the impact of land use changes at the Makhitha wetland, Limpopo province, South Africa. The data was collected using techniques such as ecological survey, socio-economic survey and interpretation of satellite images obtained between 1978 and 2004. The study findings revealed that factors such as poverty and population growth were the driving forces behind wetland mismanagement. The cultivation of crops, grazing of livestock and road construction were the main land use activities that were practised in the wetland, which contributed to wetland deterioration, a disaster that can be mitigated. The study then recommended strategies such as environmental education, fencing and land use planning to resolve the problem of land use changes, in order to contribute to sustainable wetland management.

## Introduction

Land is a finite resource where all human activities are undertaken. The main purposes of land use are building human settlements, recreation and practising agriculture such as keeping livestock and growing of vegetables and crops. Land use is of major importance to humanity such that several disciplines are focused on land issues (William & Riebsama 1993). Land use change is fundamentally a spatial process, resulting from the collective outcome of a myriad of socio-economic, institutional, biophysical and ecological processes (Wu & Wu 2013). Land use changes driven by human activity have the potential to significantly affect food security and the sustainability of the world agricultural and forest product supply systems (Popp et al. 2014). Land use change is a feature of both rural and urban areas and occurs in both developed and developing countries (Galbraith, Amerasinghe & Huber-Lee 2005; MEAB 2005). Land use change is an essential component, and it is used as an indicator of the development in an area or in a country. Wetlands are gaining recognition because of their unique contribution to aquatic ecosystems and have classified and protected according to the Ramsar Convention of 1971, articles 1.1 and 2.1 (Galbraith et al. 2005). South Africa has been a signatory to the Ramsar Convention and has enacted legislation, such as the *National Water Act* (Act 36 of 1998) and the *National Environmental Management Act* (Act 107 of 1998) to protect wetland from further destruction and degradation (Cowan 1995).

Thus, in South Africa, any development requires a permit and/or authorisation to proceed as per the *National Water Act* and the *National Environmental Management Act*. This happens after an extensive consultation period where various stakeholders within the prescribed regions debate and should be consulted. The main reasons are to avoid conflicts among the land users, and all must agree before the environmental authorisation or record of decision is issued out (William & Riebsama 1993). Accessibility plays a significant role in the location of land use. Some locations are very expensive because they have a high degree of accessibility. Land that is highly accessible has high economic value because it satisfies needs such as space for housing and other land uses, and there is always intense competition for locations (Hasan et al. 2017). Competition for land between various potential users will create the pattern of land use in a particular area (Mather 1994). In rural communities, wetlands play an important role such as the provision of water for irrigation, livestock and drinking.

Healthy, functioning wetland is vital for the protection of the environment and public. However, the majority of wetlands in South Africa are threatened because they are seen as wastelands (Dale et al. 2000). This results in the degradation and loss of wetlands because of the number of land use activities that are practised in wetland area.

For example, commercial agriculture that is carried out in south of Limpopo province in Tzaneen area and north of Mpumalanga province in Leroro area for sugar cane and tree plantation, causes a disappearance of some wetlands (Mercer 1991). The practice is to drain and destroy the vital wetland functions since sugar cane and tree plantation require a lot of water that can affect the hydrology of the area.

Another significant cause of wetland degradation and loss is urban use, which occurs in KwaZulu-Natal at Pietermaritzburg where wetlands are cleared for construction of road and industrial purposes (Mercer 1991). Also the poor use of wetland like the livestock grazing, particularly in Limpopo province, lends to wetland destruction (Dale et al. 2000). Wetlands are sometimes drained and turned into settlement purposes in order to keep up with the population growth that is increasing rapidly in South Africa. This study seeks to investigate the causes and impacts of land use changes on the wetland in Makhitha village, Limpopo province of South Africa. This research project will contribute to strategy development and better management of the wetland and avoid long-term disaster occurrence because of non-functional wetland. The specific objectives were to find out the causes of land use changes in Makhitha village, identify the major land use activities on wetland, assess the ecological impacts of land use change in the area and develop or recommend strategies that can be used to solve the problem of land use in Makhitha village.

## Materials and methods

### Characteristics of the study area

**FIGURE 1:**
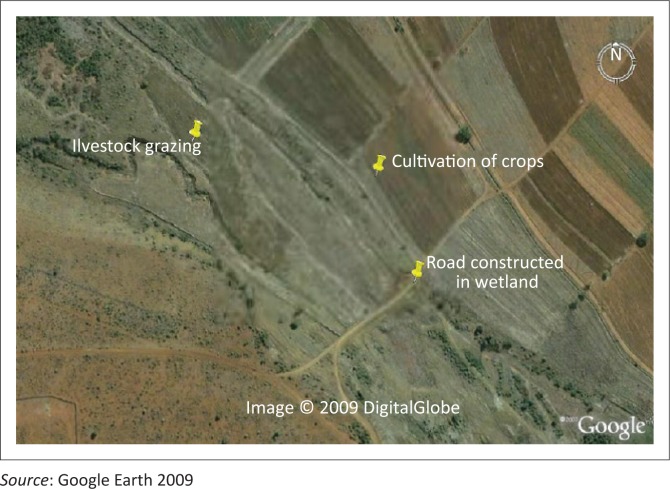
Magnified satellite image of the study area: Makhitha village, located within Makhado municipality, Vhembe district, Limpopo Province.

The study was conducted in Makhitha village, which is located in Makhado municipality under Vhembe district, Limpopo province (Figure 1). Makhitha village is situated at the foot of the Soutpansberg Mountain and lies between latitude 23° 02′ 73″ south and longitude 29° 40′ 36″ east.

The study area is dominated by sedimentary rock, mostly sandstone, shale stone and hematite (Malaza 2014). The dominant soil type in the area is loamy with high organic matter contents and high permeability. The soil colour is reddish brown, and the colour indicates that the soil is rich in nutrients and supports different types of vegetation communities and land uses. The study area is characterised by various plant and animal species. Plants range from grasses, trees, herbs and shrubs. According to Makhado Municipality (2009), the number of households was 1950 with a total population of 4134. The houses are close to each other following a dense settlement pattern.

The study area is located within a subtropical climatic region, with high temperatures in summer and low temperatures in winter. The study area has hot summer months, which usually start from September to April, and winter starts from May to August (Kephe, Petja & Kabanda 2016). The lowest temperature experienced is around 10°C in June and July, and the highest temperature is about 34°C in January (WRC 2001). The study area normally receives seasonal rainfall and the highest rainfall is received between September and March, and the lowest rainfall is received between April and July. The area receives about 400 mm–600 mm rainfall per annum (WRC 2001). The river in the study area is the Muengedzi River and the Makhitha wetland is also a part of the river. The river water is used for many purposes, such as washing clothes, cars, etc., irrigation of crops and for livestock. The land use in the study area is used for different purposes, such as human settlement, subsistence agriculture and for grazing. The greater proportion of the land use is human settlement and subsistence agriculture and reduced pastures for livestock.

### Ecological survey

The study area was visited several times to assess the existing situation. During the ecological survey, data was collected from each 100 m², and these units were approximately 20 m × 5 m in size. Some photographs of the areas of interest showing different land practices were taken. Major land use activities that were being practised in the area were also identified and captured. Condition of the wetland in the area was also observed.

### Socio-economic survey

One-on-one interviews with government officials responsible for wetland management were conducted. Three members from the Limpopo Department of Economic Development, Environment and Tourism based in the Makhado town were interviewed. Local community members who were found in the study area during field survey were also interviewed. The data obtained from interviews were concerned with the causes of wetland destruction, condition of wetland and threat to the wetland in Makhitha village.

Questionnaires were designed and administered to the people in the village. The number of participants was 195, and this was calculated as 10% of the number of households in the study area. The list of names of the households was requested from the Makhado municipality, and these names were thrown in a hat. The names were then picked at random until the 195 number was reached, and this formed the basis for the distribution of the questionnaires. Sets of questionnaires were distributed in a random manner to the villagers, to receive their views on the wetland destruction. The questionnaire was divided into two main sections. The first section collected information on the socio-economic characteristics of the respondents, for example, household monthly income, age, level of employment and gender.

The second section dealt with people’s views on the wetland. Although the sample target was 195, we managed to distribute 110 questionnaires, and only 80 filled questionnaires were received. This means that 73% of the answered questionnaires were received and usable. About 27% of the questionnaires were not returned back.

### Satellite images

Landsat satellite images of 1978 and 2004 were collected based on quality and clarity (less cloud cover), and spacing focused on vegetation cover (Table 1). Thus, the changes in vegetation cover may directly link to land use change (Naibbi et al. 2014).

**TABLE 1:** The data on satellite images.

Image type	Satellite type	Acquisition date	Resolution (m)
MSS 1978	Landsat-2	1978-11-18	60 × 60
ETM+1999	Landsat-7	1999-12-08	30 × 30

*Source:* Naibbi et al. 2014

### Data analysis

The socio-economic data were coded into an MS Excel sheet and then analysed with graphical representation. The satellite data images were analyzed qualitatively. The quantitative ideal Normalised Difference Vegetation Index (NDVI) classification as proposed by Naibbi et al. (2014) was not carried out in this study and is a major limitation of the current study.

## Results and discussion

### Factors that contribute to land use changes

There are a number of factors that contribute to land use changes, and these are population increase, poverty, use of land for agriculture and grazing. Population increase was one of the factors that were influencing land use change in the study area. Population growth increases the demand of land for food and settlement, thus leading to intensification of agriculture and expansion of cultivated land. In Makhitha village, the population increased at an alarming rate. According to Makhado Municipality (2009), in 2000 the total population was 2540 and this rose to 4134 in 2006. This high rate of population growth causes the change in land use as more households seek new land to construct houses and for agricultural purpose. This is further attested by Naibbi et al. (2014) who found that the population increase added pressure on human settlement expansion on new virgin lands, leading to land use change favouring human settlements. The expansion of human settlements in wetlands is one way of relieving pressure on existing land use change (Tian et al. 2015).

This study showed that the households had a maximum of 5–8 members (Table 2) in a family. This is similar to the study of Rananga and Gumbo (2015) who showed that larger family size was the norm in the rural communities of South Africa. Also, as these family members became older, they would seek new virgin land for their own settlement and to practise subsistence agriculture, thus exerting pressure on the available land.

**TABLE 2:** Family size of the household.

Number of persons per household	Number of respondents	Percentage
1–4	17	21
5–8	36	45
9+	27	34

Poverty is another factor that contributes to land use change. Poverty has forced people to practise subsistence agriculture (Lambin et al. 2001; Nguyen et al. 2017). The study showed that the majority of the respondents earned less than R1000 a month, which translates to US$2.57 per day (Table 3).

**TABLE 3:** Monthly income of the respondents.

Monthly income	Number of respondents	Percentage
R5000 and above	7	9
R4000–5000	11	14
R2000–3000	16	20
R1000–2000	20	24
Below R1000	26	33
Total	80	100

*Source:* Exchange rate sourced from Standard Bank (n.d.)

US$1 = R12.92.

This is just above the international poverty figure of US$1.90 per day (Ferreira, Mitchell & Beer 2015). In South Africa the poverty figure was R577 per month in 2009 (Woldesenbet et al. 2017). As a result of low or non-existence earnings, the respondents resort to subsistence agriculture. This implies that the land that is available is near or in the wetland, and the availability of water for irrigation leads to wetland degradation (Nguyen et al. 2017). The other option is to overexploit the natural resources, found in wetland, such as fish, thatching grass and sedge to make handbags, mats, hats and baskets to sell as their source of income to support their families.

### Major land use activities around the wetland

**FIGURE 2:**
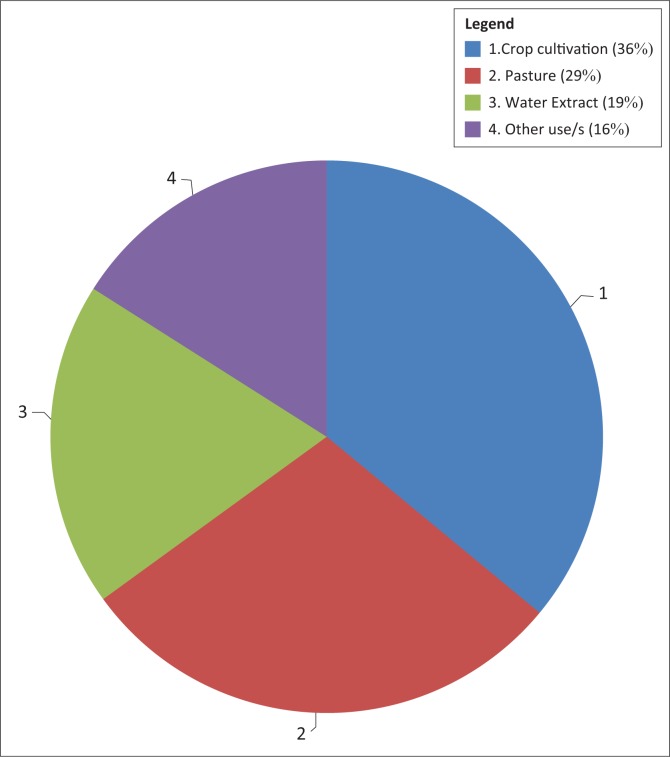
The uses of the wetland in Makhitha village.

From the ecological survey carried out in the area of study, the major land use activities were identified, which included cultivation, grazing and construction of road (Figure 2). In the Makhitha wetland, agricultural production was the major activity, with maize being cultivated by taking advantage of available water in the wetland. This means that the wetland vegetation was removed to cultivate crops during the summer season. The households used furrow irrigation in an inefficient and wasteful manner, and all this led to wetland destruction and wasting of water.

Many wetlands have been destroyed for agriculture, and agriculture is an important cause for pollution to wetlands, either by production of sediment or by generation of chemical herbicides, and these chemical wastes tend to regulate the growth of aquatic wetland plants (Goudie 2005).

Crops do not bind or cover the soil as well as the natural wetland vegetation. So by destroying the natural vegetation, soil erosion in the wetland becomes difficult to manage and control. This can be a serious problem in areas with high erosion potential. Adding fertiliser and pesticides (which may leach into the river system) further reduces the effectiveness of the wetland in purifying water. The amount of organic matter in the soil may also be decreased (Low & Rebelo 1996). Wetland served as a source of water and food to domestic animals, particularly during the winter period. There was no limitation on the number of livestock. Twenty-nine per cent of respondents indicated that they used wetland for grazing, and most of them own more than 10 cattle, usually goats and donkeys. This has led to over-grazing of wetland vegetation and caused erosion of fertile soil around the wetland. This caused the wetland to lose value to regenerate the vegetation, and these result in loss of biodiversity (Marty 2015). For example, vegetation such as Cattails (*Typha*), Bulrushes (*scirpus*) and Spike Rushes (*Eleocharis*) decreased. Wetlands, especially temporarily and seasonally waterlogged areas, may provide valuable grazing fields for domestic and wild grazers. This particularly happens in the early growing season and during droughts when grazing reserves are low in the surrounding veld (rangeland), but the wetlands continue to produce a lot of grazing (Braack, Walters & Kotze 2000). The other 16% of respondents used the wetland for harvesting of reeds and sedges for making handcrafts in South Africa, such as the *Juncos kraussii* (Incema) and the sedges (*Cyperus latifolius*) (Ikhwane and *Cyperus textilis*). The common reed (*Phragmites australis*) is used for construction purposes. Some wetland plants are also collected for their medicinal properties (Braack et al. 2000).

**FIGURE 3:**
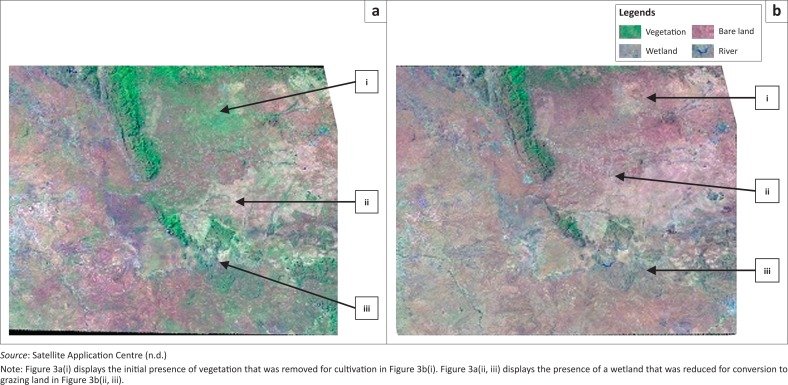
Satellite images of land use changes in the study area for (a) 1978 and (b) 2004.

A large portion of the wetland in the study area was used as a path and a road (Figure 3). This was because of the lack of a bridge that can cross the Tshipata River. During the ecological survey, a man from the area who was interviewed said that a road was constructed so that they could cross the wetland more easily without any inconvenience. The construction of infrastructure such as roads and houses in wetland is not a new activity as this has been practised world over, leading to wetland destruction and loss of biodiversity (Malekmohammadi & Blouchi 2014). Interviews with the Limpopo Department of Economic Development, Environment and Tourism officers indicated that according to the *Limpopo Environmental Management Act* (no. 3 of 2003) no person was allowed to construct a road in a wetland (Department of Agriculture 2006) unless the person had permission to do so, because a road in a wetland would disturb its normal functioning.

### Progress in the wetland encroachment

Satellite images were analysed. Figure 3 shows the land use change between 1978 and 2004. The land use change that occurred was probably because of grazing, crop cultivation, etc. Vegetation can be seen in Figure 3a(i), whereas in Figure 3a(ii), there is a huge wetland.

After the different land use changes that were derived by human activities, the vegetation that can be seen in Figure 3a(i) was removed for cultivation purposes, as can be seen in Figure 3b(i). Wetland showed in Figure 3a(ii, iii) was reduced and such land was converted into grazing land for livestock, which is shown in Figure 3b(ii, iii). Wetlands are one of the richest ecosystems in terms of biodiversity; they rank right up there with rainforests – although many wetland species are fewer and go unnoticed (Kotze et al. 2000). In the study area, wetland is no longer covered with vegetation because of different human activities. These activities include agriculture, grazing and transportation. Different types of plants that were found within the study area are no longer found because plant species are used for food and shelter, and this causes wetlands with fewer animals and plants in the study area.

The plants, in turn, provide food and shelter for animals. Several wetlands species, such as the white-wing flufftail and wattled crane, are listed as Red Data species (Williams 1990). Intact wetlands could help us combat global warming. In wetlands, the decomposition of organic matter is slowed down by the anaerobic conditions present. This results in wetlands trapping carbon as soil organic matter instead of releasing it into the atmosphere as carbon dioxide. Presently too much carbon dioxide is being released into the atmosphere when fossil fuels (such as coal and oil) are used to produce energy, resulting in the global climate being disrupted. Coal is, in fact, formed from plant material accumulated under wetland conditions in swamps that existed millions of years ago. Thus, instead of destroying wetlands and releasing carbon dioxide into the atmosphere, we should conserve them (Braack et al. 2000).

## Conclusion

Wetlands are vital for the protection of the environment and public health; unfortunately, they are not well understood. Wetlands perform functions that have positive environmental influence and social and economic significance. For example, wetland manages water for free by holding backwater during floods and releasing it during dry periods. In a dry country like South Africa, this is crucial and helps to prevent soil erosion.

Although wetlands are important for people’s life and environment, there are a number of land use activities that are still practised which have adverse negative impacts. In some areas where a wetland is found, there are no strategies implemented to solve or mitigate the impacts of different land use activities. In some other areas, very little is done to minimise the problems of land use change on wetlands. It is therefore important that local communities take action on the management of wetlands ecosystem.

### Recommendation

Fencing is also one of the essential management strategies that can be used to protect the wetland in Makhitha village so that these wetlands will not be destroyed by random land use changes that cause wetland destruction in the study area. A fence is required to cover the entire wetland area. It needs effective planning, involving both the community and government to encourage the productive use of the wetland without destroying it. The advantages are that the implementation of fence is easy and may create employment for local community. This strategy allows the wetland vegetation to be grown effectively without any disturbance. The cattle that graze in wetland can be controlled so that there will be no longer any over-grazing.

Environmental education is the way of educating people about environment. In this case, people need to be educated on the importance of the wetland. This strategy can help reduce the problem of land use change in and around the wetland. People have to be aware that wetland provides many benefits which have important values to the community. For example, plants that are found within wetland provide oxygen which is crucial for the survival of humans. Wetland plants also help prevent the problem of soil erosion. In a country like South Africa with scarcity of water, it provides water for domestic and commercial purposes. This has to be done by the Department of Environmental Affairs in collaboration with the Department of Water Affairs and Forestry. The targeted group for environmental education should be pre-school, primary and secondary schools and at workplace. Environmental education can also be spread through radios, televisions and newspapers.

## References

[CIT0001] Braack A.M., Walters D. & Kotze D.C., 2000, *Practical wetland management*, Rennies Wetlands Project, n.p.

[CIT0002] Cowan G.I., 1995, ‘South Africa and the Ramsar Convention’, in Cowan G.I. (ed.), *Wetlands of South Africa*, Department of Environmental Affairs and Tourism, Pretoria.

[CIT0003] Dale V.H., Brown S., Haeuber R., Hobbs N.T., Huntly N., Naiman R.J. et al., 2000, ‘Ecological principles & guidelines for managing use of land’, *Ecological Application*, 10(3), 639–670. 10.2307/2641032

[CIT0004] Department of Agriculture, 2006, *Statistics of weather condition Limpopo Province, Makhado*, Government Gazette 2643, Government Printers, Pretoria.

[CIT0005] Ferreira F., Mitchell D. & Beer P., 2015, *The international poverty line has just been raised to $1.90 a day, but global poverty is basically unchanged. How is that possible?*, viewed n.d., from https://blogs.worldbank.org/developmenttalk/international-poverty-line-has-just-been-raised-190-day-global-poverty-basically-unchanged-how-even.

[CIT0006] Galbraith H., Amerasinghe P. & Huber-Lee A., 2005, *The effects of agricultural irrigation on wetland ecosystems in developing countries: A literature review*, Comprehensive Assessment Secretariat, Colombo.

[CIT0007] Google Earth, 2009, *Makhitha Village*, viewed n.d., from https://earth.google.com/web/@-23.0521495,29.67845295,851.65398984a,5139.06304535d,35y,0.0000001h,0t,0r/data=ChUaEwoLL2cvMXZ5cjgzMnMYAiABKAI.

[CIT0008] Goudie A., 2005, *The human impact on the natural environment*, Blackwell, Oxford.

[CIT0009] Hasan S., Wang X., Khoo Y.B. & Foliente G., 2017, ‘Accessibility and socio-economic development of human settlements’, *PLoS One* 12(6), e0179620 10.1371/journal.pone.0179620 28636630 PMC5479555

[CIT0010] Human Sciences Research Council (HSRC), 1997, *Code of research ethics*, HSRC, Cape Town.

[CIT0011] Kephe P.N., Petja B.M. & Kabanda T.A., 2016, ‘Spatial and inter-seasonal behaviour of rainfall in the Soutpansberg region of South Africa as attributed to the changing climate’, *Theoretical and Applied Climatology* 126(1–2), 233–245. 10.1007/s00704-015-1569-9

[CIT0012] Kotze D.C., Breen C.M. & Klug J.R., 2000, *A wetland management decision support system for South Africa freshwater palustrine wetland*, 3rd edn., Department of Environmental Affairs & Tourism, Pretoria.

[CIT0013] Lambin E.F., Turner B.L., Geist H.J., Agbola S.B., Angelsen A., Bruce J.W. et al., 2001, ‘The causes of land-use and land-cover change: Moving beyond the myths’, *Global Environmental Change* 11(4), 261–269. 10.1016/S0959-3780(01)00007-3

[CIT0014] Low A.B. & Rebelo A.G. (eds), 1996, *Vegetation map of South Africa: Lesotho and Swaziland*, Department of Environmental Affairs & Tourism, Pretoria.

[CIT0015] Makhado Municipality, 2009, *Makhado Municipality integrated development plan*, Makhado Municipality, Makhado.

[CIT0016] Malaza N., 2014, ‘Basin analysis of the Soutpansberg and Tuli coalfields, Limpopo province of South Africa’, PhD dissertation, University of Fort Hare.

[CIT0017] Marty J.T., 2015, ‘Loss of biodiversity and hydrologic function in seasonal wetlands persists over 10 years of livestock grazing removal’, *Restoration Ecology* 23(5), 548–554. 10.1111/rec.12226

[CIT0018] Mather A.S., 1994, *Land use*, Longman Group, New York.

[CIT0019] Mercer D.C., 1991, *Recreation and wetland impacts, conflict and policy issues In Williams wetland a threatened landscape*, Blackwell Inc., Cambridge.

[CIT0020] Malekmohammadi B. & Blouci L.R., 2014, “cological risk assessment of wetland ecosystems using multi criteria decision making geographic information system’, *Ecological Indicators* 41(1), 133–144. 10.1016/j.ecolind.2014.01.038

[CIT0021] Millennium Ecosystem Assessment Board (MEAB), 2005, *Ecosystems and human well-bing: Wetlands and water synthesis*, World Resources Institute, Washington, DC.

[CIT0022] Naibbi A., Baily B., Healey R.G. & Collier P., 2014, ‘Changing vegetation patterns in Yobe State Nigeria: An analysis of the rates of change, potential causes and the implications for sustainable resource management’, *International Journal of Geosciences* 5(1), 50–62. 10.4236/ijg.2014.51007

[CIT0023] Nguyen H.H., Dargus P., Moss P. & Aziz A.A., 2017, ‘Land-se and socio-ecological drivers of wetland conversion in Ha Tien Plain, Mekong Delta, Vietnam’, *Land Use Policy* 64(1), 101–113. 10.1016/j.landusepol.2017.02.019

[CIT0024] Popp J., Lakner Z., Harangi-Rákos M. & Fári M., 2014, ‘The effect of bioenergy expansion: Food, energy, and environment’, *Renewable and Sustainable Energy Reviews* 32(C), 559–578. 10.1016/j.rser.2014.01.056

[CIT0025] Rananga H.T. & Gumbo J.R., 2015, ‘Willingness to pay for water services in two communities of Mutale Local Municipality, South Africa: A case study’, *Journal of Human Ecology* 49(3), 231–243. 10.1080/09709274.2015.11906841

[CIT0026] Standard Bank, n.d., *Foreign exchange*, viewed 27 June 2017, from https://www.standardbank.co.za/southafrica/personal/products-and-services/bank-with-us/foreign-exchange.

[CIT0027] Tian B., Zhou Y.X., Thom R.M., Diefender H.L. & Yuan Q., 2015, ‘Detecting wetland changes in Shanghai, China using FORMOSAT and Landsat TM imagery’, *Journal of Hydrology* 529(1), 1–10. 10.1016/j.jhydrol.2015.07.007

[CIT0028] Water Research Commission (WRC), 2001, *State of rivers report: Letaba and Luvuvhu river systems, WRC Report, Number TT 165/01*, Water Research Commission, Pretoria.

[CIT0029] William P. & Riebsama W., 1993, *Land, food and rural development in North Africa*, West View Press, San Francisco, CA.

[CIT0030] Williams L.C, 1990, *The Greater St Lucia Wetland Park*, 1st edn., National Parks Board, Pretoria.

[CIT0031] Woldesenbet S.A., Jackson D.J., Lombard C.J., Dinh T.H., Ramokolo V., Doherty T. et al., 2017, ‘Structural level differences in the mother-to-child HIV transmission rate in South Africa: A multilevel assessment of individual-, health facility-, and provincial-level predictors of infant HIV transmission’, *Journal of Acquired Immune Deficiency Syndromes* 74(5), 523 10.1097/QAI.0000000000001289 28107227 PMC5351751

[CIT0032] Wu J. & Wu T., 2013, ‘Ecological resilience as a foundation for urban design and sustainability’, in Pickett S.T.A., Cadenasso M.L. & McGrath B. (eds.), *Resilience in ecology and urban design*, pp. 211–229, Springer, Dordrecht.

